# Clinical study comparing the accuracy of interocclusal records, digitally obtained by three different devices

**DOI:** 10.1007/s00784-022-04542-6

**Published:** 2022-05-18

**Authors:** Cristina Fraile, Alberto Ferreiroa, Marta Romeo Rubio, Raquel Alonso, Guillermo Pradíes Ramiro

**Affiliations:** 1 Department of Conservative and Prosthetic Dentistry, Faculty of Odontology, University Complutense of Madrid, Plaza Ramón y Cajal S/N, Ciudad Universitaria, 28040 Madrid, Spain; 2 Department of Conservative and Prosthetic Dentistry, Complutense University of Madrid, Madrid, Spain

## Introduction

Obtaining a reliable occlusion register with appropriate occlusal contacts is considered of great importance when restoring patients in dentistry workflows [1]. In prosthetic and restorative procedures, to obtain an accurate record and to transfer this occlusion to the dental technician play an important role [2].

Traditionally, articulating papers with different thicknesses as well as shim stock (8-μ aluminium foil) have usually been adopted as a standard to analyse the occlusal contacts directly in patients or even in plaster models. According to the reviewed literature, the occlusal tactile sensibility for natural teeth can be as low as 8–10 μm; thus, the 8-μm articulating paper seems to be more appropriate than other thicknesses such as 40 or 200 μm [3–8]. For this reason, it is frequently considered the gold standard thickness to correctly detect interocclusal contacts.

However, articulating paper presents some limitations: patients need to bite several times to obtain full arch contacts; saliva can also interfere on results promoting the creation of false positives and false negatives and intensity; and finally, the bite sequence cannot be analysed. However, none of these techniques has been scientifically proven to be an ‘ideal’ method to analyse the occlusion [3].

During the last 10 years, digital impressions obtained by using chairside intraoral scanners have been increasingly implemented. This information is based on the use of maxillary and mandibular digital models in STL format (Standard Triangle Language). In order to obtain a correct interocclusal spatial position of the maxilla and mandible files articulated, a 3rd file of the buccal view of the intermaxillary articulation is done by using reference points processed with a mathematical algorithm [4–6]. The correct simulation of patient’s occlusal contacts is needed to set virtual models in the desired intercuspation position by the operator (normally maximum intercuspal position or centric occlusion). This register allows us to omit an interocclusal record by using elastomers and after that scanning this record. Thus, concerns about dimensional stability of interocclusal record materials are eliminated, and the interocclusal record process is simplified [2]. The advantages of using Computer Aid Impression (CAI) can be summarised in the elimination of clinical time-consuming steps, enhanced patient comfort and data process stored, the elimination of laboratory time to pour and pin models, inaccuracies in handing trimming and finally, no need of using mechanical articulators and facebow [7].

The development of digital methods to record occlusal contacts, like T-Scan III system (Tekscan, Boston, MA, USA) has allowed dentistry procedures to be more comfortable, faster and with higher quality possibilities [8, 9]. T-Scan III system is the cutting edge version of this digital occlusal indicator device. It can analyse and report occlusion in terms of the sequence of each tooth contact, force location on the contacting tooth surfaces, relative occlusal force in percentage values and centre of force trajectory. Although data from T-Scan III are more precise and quantitative than conventional occlusal indicators, T-Scan III is not prevailingly used due to its high cost. For this reason, patient and clinician´s perceptions are still key factors to validate occlusion [10].

Great differences have been found when analysing and recording occlusal contacts in in vivo studies compared with in vitro studies. In in vitro studies, it is easier to analyse the different variables of occlusal contacts, like contact areas or intensity of occlusal contacts. Regarding in vivo studies normally, data obtained have to be reduced to the most simple level of interpretation due to the large number of variables that can affect and bias the real contact points, so in the end it can easily be misinterpreted [3, 5].

The aim of this cross-sectional study was to compare the reliability of interocclusal contact records obtained by using conventional methods with digital methods using intra- and extraoral digital scanners and T-Scan III system and by conventional method directly in patients (8-μm articulating paper). The null hypothesis established that there were no differences between the teeth ubication of contact points obtained with conventional and digital methods.

## Materials and methods

### Subjects

Twenty-five healthy volunteers (5 men and 20 women) were selected. The number of patients was previously calculated with the software G* Power 3.1 for an expecting power of 80% and alpha value of 0.05. Formula results suggest using a minimal of 22 patients. At the beginning of the study, 30 patients were included and after the dropping out of 5 patients during the timeline of the study, finally 25 patients were used for the statistical analysis. The inclusion criteria of this cross-sectional study were as follows: not missing teeth, no need for dental treatments, occlusal stability, not bruxism patients and minimal or not occlusal wear. The presence of temporomandibular joint disorders, anterior or posterior open bite and not signing the informed consent were considered exclusion criteria. Ethical approval was granted by the Ethics Committee (16/273-E. Hospital Clínico San Carlos). All volunteers were informed about the objectives of the study and signed the corresponding informed consent form. All the study was conducted in accordance with the Declaration of Helsinki and Good Clinical Practice.

### Control group

To determine the physical occlusal contact locations, patients were asked to bite in maximum intercuspation and 8-μ articulating papers were used to verify the contacts (8μ Arti-Fol, Dr. Jean Bausch GmbH & Co.). Contacts were confirmed with shimstock foils (Hanel Shimstock, Roeko Dental, Langenau, Germany). A set of intraoral photographs were taken in order to register the contact points. These were considered the gold standard for this study (Fig. 1).

### Digital group

**Fig. 1:**
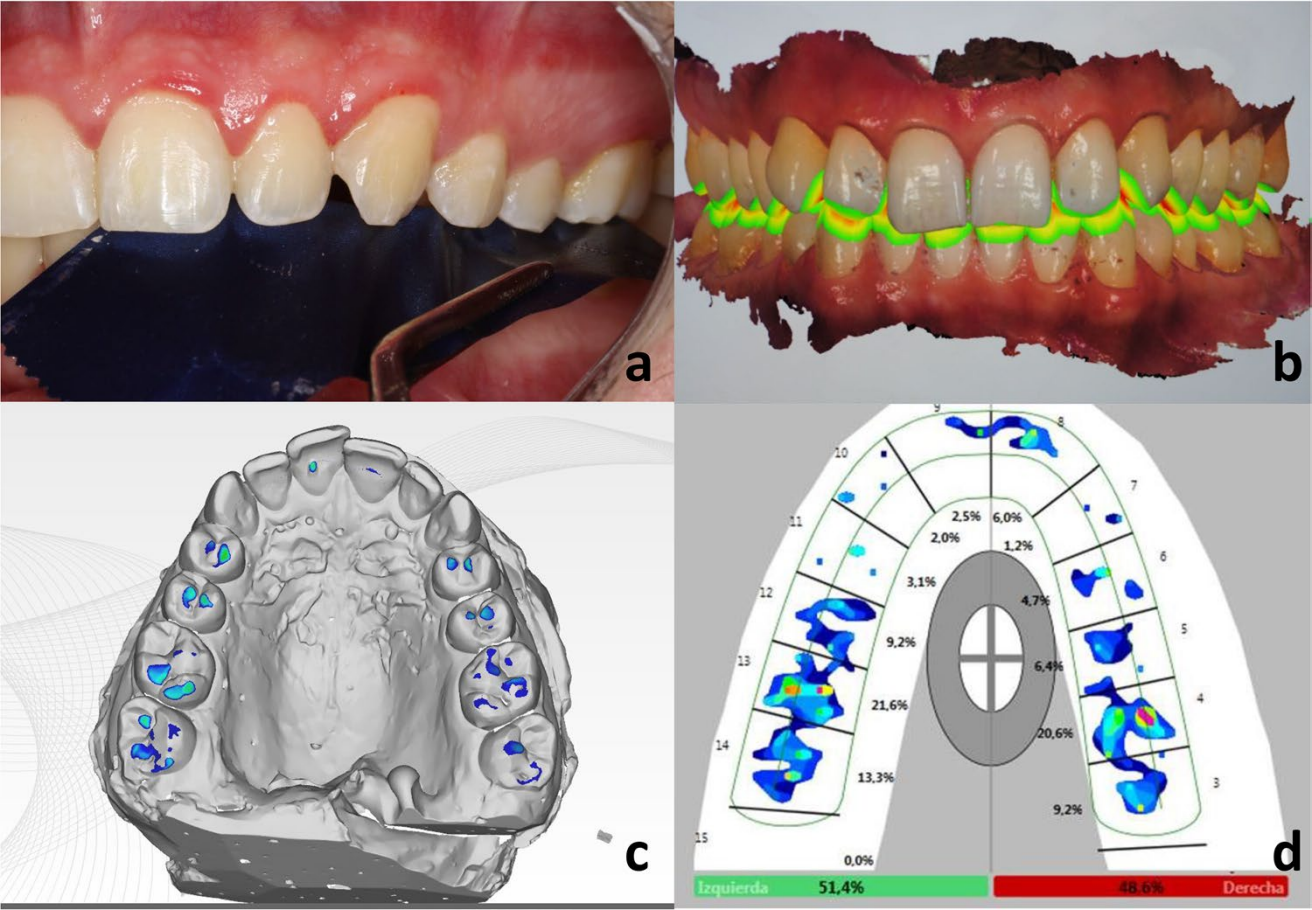
**a** Control group. **b** Intraoral scanned. **c** Extraoral scanned. **d** T-Scan III record of patient n° 17.

#### Intraoral scanner

Digital impressions of the upper and lower complete arches, as well as two interocclusal vestibular records from every patient, were made with an intraoral scanner (Trios Color POD, Phibo, 3 Shape), according to the manufacturer instructions and recommendations. This information was stored for further analysis.

#### Extraoral scanner

Later on, for every patient, a single step double mix silicone consistency (Virtual, Ivoclar Vivadent, Schaan, Liechtenstein) impression was taken and poured in type IV plaster (Fujirock, GC Europe, Leuven, Belgium). Then, after using the facebow, upper and lower models were mounted in a semi-adjustable articulator in maximal intercuspal position (Stratos 300, Ivoclar Vivadent). Afterwards, plaster models were scanned with an extraoral scanner (Zfx Evolution, Zimmer Biomet Dental, Inc. Ca, USA) and the upper and lower models were aligned with the lower intercuspation position in order to analyse and compare the occlusal contacts (Zfx Manager, Design Cad 6.0).

#### T-Scan III

Finally, all intermaxillary records were made with the T-Scan III occlusal analysis system (Klockner, Tekscan Inc., South Boston, Mass) software 9.0 (v9.1.11). The width of the central incisor and absence or presence of wisdom teeth were individualised in the file for each patient. To register the T-Scan III, it was necessary to instruct the patient on how to perform the opening and closing movements.

### Statistical analysis

Two previously trained and calibrated examiners recorded independently the interocclusal contacts for all the different groups, classifying them in a dichotomy variable (yes/no) contact or not per tooth, regardless of the surface amount or contact intensity. To consider a mark as an occlusal contact, two examiners registered the contacts at the earliest moment in which an occlusal colour mark could be optically visualised. All data was stored and analysed using SPSS 19.00 software for Mac (IBM).

Descriptive results expressed for each type of interocclusal records were analysed using Pearson’s chi-squared test and Cohen’s kappa coefficient. Subsequently, the diagnostic tests (sensitivity, specificity and predictive values for positives and negatives) as well as receiver operating characteristic (ROC) curve was performed.

## Results

When gold standard contacts (8-μ articulating paper contact group) registered by two examiners were compared, kappa interrater index was 70.6%. This agreement was considered ‘*better*’ (Table 1).

**Table 1:** Kappa index results

	Kappa index	Agreement
Intraoral scanner	56.1%	Moderate
Extraoral scanner	50.3%	Moderate
T-Scan III	29.9%	Low

When the intraoperator kappa index was performed (8-μ articulating paper contact group was the gold standard), greatest agreement was obtained in the intraoral scanner contact group (56.1%, *moderate* agreement) and worst value in the T-Scan III contact group (29.9%, *low* agreement) (Table 2).

**Table 2:** Diagnostic test results using the 8-μ articulating paper as gold standard

Variables	Sensitivity	Specificity	PPN	NPV
Intraoral scanner	83.82%	79.48%	93.44%	58.49%
Extraoral scanner	90.80%	57.69%	88.21%	64.28%
T-Scan III	98.16%	24.35%	81.90%	79.16%

Regarding diagnostic tests results, sensitivity results were between 83.82 and 98.16%, obtaining the lowest sensitivity values in the intraoral scanner group and highest values in the T-Scan III contact group. Specificity results range from 24.35 to 79.48%. Highest specificity values were obtained by an intraoral scanner and lowest values were obtained in the T-Scan III contact group. Positive predictive value results range from 81.90 to 93.44%, being the highest value for the intraoral scanner. Finally, highest negative predictive value results were obtained in the T-Scan III contact group (79.16%).

**Fig. 2:**
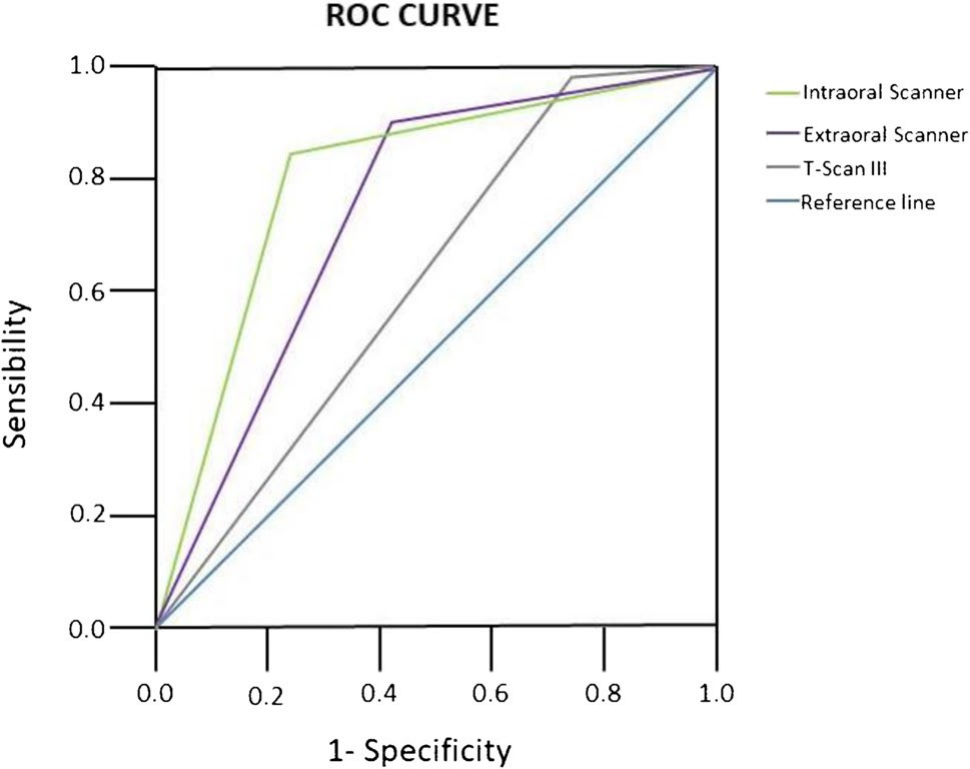
ROC curve results

ROC curve results are shown in Table 3 and in Fig. 2.

**Table 3:** ROC curve results and significance

Intraoral scanner	.817	.0001
Extraoral scanner	.743	.0001
T-Scan III	.613	.002

## Discussion

The null hypothesis of the study established that there were no differences between contacts obtained with conventional and digital methods. From the obtained results, the null hypothesis can be rejected, because statistical significant differences were obtained between the contact groups, being the intraoral scanner group having the better results.

Over the years, several materials and methods have been mentioned to record occlusal contacts in the scientific literature. Despite the development in the study of occlusion, it is necessary to investigate and develop more and new objective methods than those already existing [11].

For this study, the conventional contact group (articulating paper) was compared with the digitally contact groups. To obtain the control or standard contact group, two examiners registered occlusal contacts, and to validate this measurements, kappa interrater index was made and was considered *better* agreement (70.6%). Several studies chose the same method to register the contacts of the gold standard group [2, 5, 6, 12, 13].

The kappa index results from the current in vivo study show the best agreement in the intraoral scanner contact group (56.1%, moderate) but the extraoral scanner contact group presents almost the same agreement (50.3%, moderate). The diagnostic test results also demonstrated better results in the intraoral scanner contact group. Some authors (Delong et al., 2002) establish minimum values to confirm a new method as a reliable diagnostic test; these are results in sensitivity greater than 0.70 and in specificity greater than 0.90 [5]. In the current study, all sensitivity’s results were higher than 0.70 but not specificity’s results. According to this, only the intraoral scanner contact group can approach to be a reliable method to analyse the occlusal contacts.

As regards the ROC curve results, all curves were situated in the upper left region of the diagonal line. The AUC (area under the curve) value was 0.817 in the intraoral scanner contact group, which indicates that intraoral scanner has excellent discriminating power in the detection of the occlusal contacts. The AUC value in the extraoral scanner contact group was 0.743, which indicates that extraoral scanner has acceptable discriminating potential in the detection of the occlusal contacts.

Several studies have investigated the reliability of digital occlusal contacts. Straga compared occlusal contacts registered with 21-μm articulating paper in stone models, with those digitally obtained by extraoral scanner. We did not find concordance in the results, in terms of diagnostic test (sensitivity 54% (Straga)–90% (our results), specificity 98% (Straga)–57% (our results), PPV 76% (Straga)–88% (our results), NPV 96% (Straga)–79% (our results)). These differences can be explained due to the use of the different gold standard considered in these studies. Straga used the stone models as standard contacts. It is necessary to set a gold standard in order to analyse and to compare the occlusal contact different results of the studies. Furthermore, it is necessary to point out that in Straga’s study different extraoral scanners were used, both with optical technology [12].

Current literature includes limited information on the accuracy of occlusal contacts calculated by CAD software in virtual models or CAD/CAM restorations. To the best of the authors’ knowledge, no study has reported accuracy of virtual occlusal contacts calculated from three different devices. Delong et al. reported that accurate occlusal contacts could be calculated from aligned virtual casts with different alignment methods [5, 13]. Nemli and cols. revealed in 2012 that the accuracy of digital occlusal contacts may depend on the CAD software used [2]. Gintaute and cols. compare the precision of maxillo-mandibular registration and resulting full arch occlusion produced by three intraoral scanners (Planmeca, Cerec and Trios) and concluded that TRIOS showed more uniform occlusions and produced occlusions which were closest to the true value [14]. Recently, Abdulateef et al. concluded that the accuracy of the interocclusal contacts obtained with virtual occlusal scans was clinically acceptable. However, they used as a gold standard contacts obtained by a polyvinyl siloxane interocclusal records analysed by transillumination [15].

In literature, several studies compare occlusal contacts registered by T-Scan system with conventional contacts and accuracy’s results also were often contradictory. In the current study, the occlusal contacts obtained by conventional methods were compared with those recorded by the T-Scan III system. In our study, this group was the one with the worst results registered. This can be explained because of the thickness of the articulating paper (8 μm) used as a gold standard being significantly lower than that of the T-Scan sensors (250 μm) and this can influence on the results, generating false positives, as well as errors when obtaining the registration [16]. On the contrary, there are several studies such as Gummus and Da silva that concluded that T-Scan results are better and more reliable [3, 16].

Solaberrieta et al. in 2017 describe a novel technique based on reverse engineering technology to locate occlusal contacts with an intraoral scanner and the T-Scan III occlusal analysis system, as they claim that the T-Scan III alone offers contradictory results, in terms of repeatability and accuracy, and that further studies are needed to validate the reliability and repeatability of these digital systems [17].

In recent years, some authors have researched the reliability of occlusal contacts obtained by different intraoral scanners. They concluded that interocclusal distortions for the different intraoral scanners’ IOS were significantly different. The intraoral scanner Trios Color performed the best results in this investigation. The distortions observed by these authors will affect the magnitude of occlusal contacts of restorations clinically. The final restoration may be either hyperoccluded or infraoccluded, requiring compensations during the CAD design stage or clinical adjustments at issue [18].

This present study has some limitations. First, intraoral occlusal contacts were compared with those scanned in plaster casts, but a rigid cast cannot represent the biological environment of the oral cavity. Furthermore, in a digital intraoral impression when patients occlude in maximum intercuspation, teeth have an intrusive capacity. The intrusion of the teeth cannot be simulated in the stone models scanned, so there may be differences when compared with the intraoral situation [2]. Another limitation of this study could be the accuracy of the extraoral scanner used compared with the one obtained for coordinate measurement machine devices (CMM) used in in vitro studies.

Also, to analyse the reliability of the digital occlusal contacts, it is necessary to compare with a gold standard or a validated method. Unfortunately, a gold standard universally accepted method does not exist to identify the clinical occlusal contacts [11–17].

## Conclusions

Within the limitations of the present study, results suggest greater reliability to record occlusal contacts with digital methods using the intraoral scanner.
